# Traité des Nerfs et de leurs Maladies

**Published:** 1781-02

**Authors:** 


III. *Traité des Nerfs et de leurs Maladies. Par M.* Tiſſot, M.D. *&c.* (*Continued from Page* 51.)

THE third part of M. Tiſſot's work relates to the pathology and treatment of diſorders of the nerves. In the firſt ſection he preſents us with the opinions of different writers on this ſubject, and obſerves, that till M. Pommes's book made its appearance, nervous diſeaſes were almoſt univerſally conſidered as the effects of relaxation. This writer, whoſe work has been tranſlated into Engliſh by Dr. Berkenhout, aſcribes them to a very oppoſite cauſe, to what he he calls a *racornissement* of the nerves, and upon this principle it is that he propoſes to treat them with warm bathing and other means of relaxation. Notwithſtanding the eulogium beſtowed on this new theory by M. Tiſſot, we own we have always conſidered it as the fruit of a lively imagination, rather than of ſound reaſoning; and indeed our author acknowledges that affections of the nerves often originate from very different cauſes.

In the ſecond ſection M. Tiſſot treats of the diſeaſes peculiar to the nerves, and theſe, he thinks, may in a great meaſure depend on changes that take place in the nervous fluid. It may be too viſcid or too rare, poſſeſſing too great or too little acrimony. He apologizes for ſpeaking in this manner of the properties of this fluid, by ſhewing that although it eſcapes our ſenſes, it muſt certainly have a certain degree of conſiſtence, liable to vary, and of courſe to produce diſeaſe; and although it affords nothing that can ſtimulate our palate, or our noſtrils, yet it muſt poſſeſs a certain ſtimulating power perceptible by the muſcles, and this power may be in too great or in too weak a degree. A number of facts he obſerves prove to us, that it is ſometimes much ſtronger than at others, and of courſe he thinks it evident that the nerves near their origin are ſuſceptible of a variety of diſeaſes; and that the diſeaſes of the brain muſt in a certain degree be thoſe of the nerves themſelves, ſince the whole medulla oblongata, and probably the whole of the medullary ſubſtance, are nerves.

To theſe cauſes which may diſturb the functions of the nerves, and which are ſeated in the nerves themſelves, our author has thought proper to add other proximate cauſes, before he proceeds to examine each ſeparately: theſe are, 1. An affection of the ſenſorium. 2. A diſeaſe of the coverings of the nerves. 3. Affections of the parts ſurrounding the nerves, by which their action is diſturbed. 4. Morbid irritability.

Speaking of the remote cauſes of nervous diſeaſes, our author obſerves, that it is in countries where the air is the moſt humid, the vegetables the moſt watery, and where the greateſt uſe is made of fat and oily aliment and hot liquors, that theſe complaints are the moſt frequent.

" If we attend, ſays he, to the ſeaſons, we

" ſhall find that it is during the great heats that

" relax, or in the rainy ſeaſons that moiſten, or

" above all, during the hot ſoutherly winds

" which

" which relax and moiſten at one and the " ſame time, that theſe evils abound the moſt. " Conſult thoſe who have experienced the ef- " fects of the *Sirocco*, and they will aſſure you " that their feelings were exactly ſimilar to thoſe " attending the moſt cruel nervous hypochon- " driaſis; but that the moment a drying north- " erly wind ſet in, their complaints ceaſed. I " had for ſeveral years under my care an ex- " tremely vapouriſh woman, who in a heavy, " damp air, that was never refreſhed by a " northerly wind, was unable to move an hun- " dred yards without having an hyſteric fit, but " who in a keen, dry air could walk a league " without any inconvenience; when the north " wind blowed, ſhe uſed to ſtop that ſhe might " breathe it better, for ſhe was ſenſible that it " gave her ſtrength, and rendered her cheerful " and happy. If we compare the caſe of this " patient with that of another woman, who was " never ſo well as when ſurrounded by a relax- " ing vapour, I preſume that theſe two facts " are ſufficient to prove, that the ſame nervous " ſymptoms may depend on cauſes that are " diametrically oppoſite.

" A robuſt, healthy man, of a dry fibre, in- " ured to hard labour, and the uſe of ſtrong

" liquors, is a ſtranger to diſorders of the " nerves; no cauſe, either moral or phyſical, " can excite in him the ſymptoms that charac- " terize them; but ſuppoſe ſuch one to have " an inflammatory fever, we bleed him, recom- " mend warm bathing, confine him to a regi- " men of almond milk, barley water, chicken " broth, and light farinaceous food; preſcribe " clyſters, fomentations, &c. and at the end of " a few weeks his blood is impoveriſhed, his " nerves are relaxed, ſo that this ſtout, robuſt " man reſembles an hyſterical woman; any " particular ſmell, any thing that alarms or " ſurprizes him, the leaſt error in diet, either " of theſe excites in him all the ſymptoms " of hyſterics, ſuch as tremor, palpitation, fear, " flatus, watery urine, &c. The fact is, you " have relaxed him, and you have rendered him " hypochondriacal." Our author adds, that violent paſſions, or great loſſes of blood, have in a ſhort time produced ſimilar effects. After all, however, he is of opinion, that although there are caſes in which there is an increaſe of denſity and dryneſs in the nervous fibres, by means of which their functions are diſordered, yet that this ſtate is much leſs frequent than that of atrophy, and tends rather to produce melancholia and mania than hyſteria.

In

In treating of the diſeaſes of the coats of the nerves, M. Tiſſot, with his uſual induſtry, has collected from authors a great variety of inſtances of diſeaſe occaſioned by collections within their cellular ſubſtance, or by tumors compreſſing particular nerves, &c.

He next proceeds to treat of the prediſpoſing and occaſional cauſes of nervous diſeaſes, and begins with thoſe which proceed from any conſtitutional defect. A prediſpoſition from this ſource he is convinced may be hereditary, and he conſiders this melancholy tranſmiſſion of diſeaſe from one generation to another, as one of thoſe truths of which no one doubts, but thoſe who are determined to doubt. He quotes ſeveral inſtances from authors in proof of what he advances on this ſubject, and among others, ſpeaks of a family in which Lanciſi had occaſion to obſerve a dilatation of the right auricle and ventricle in four generations, and which produced the ſame ſymptoms in the great grandfather, grandfather, father, and ſon.

On the ſubject of air, our author obſerves that ſuch climates as are dry, and rather warm than cold, are in general the moſt favourable to the nerves. Moiſture, eſpecially when combined with heat, heat, seems to become one of the most active predisposing causes. Huxham found that in wet seasons nervous diseases were the most frequent; Viridet observed, that during the excessive hot summer of 1706, they were unusually prevalent. Zimmerman has often had occasion to observe, that during the great heats hysterical patients are affected, seemingly from no other cause, with syncope, diarrhœa, and convulsions, which disappear when the air becomes cooler. Dodart in the *Mem. de l'Acad. des Sciences* speaks of a young man who constantly lost his understanding during the hot months.

Cold, our author observes, is likewise often a powerful occasional cause. Hippocrates long ago remarked, that cold air applied to the bare nerves, sometimes excited convulsions; Galen has seen it occasion apoplexy and tetanos; similar effects have been observed from it in the northern parts of Europe; and even on the Malabar Coast, a wind sometimes blows, the coldness of which is so excessive as to produce violent convulsions. Zimmerman had the care of an hysterical patient, who, merely by exposure to cold, fell into convulsions; and M. Tissot himself has often had occasion to observe persons of irritable irritable habits affected by a ſpaſm of the whole ſurface of the body from coldneſs of the feet.

Speaking of errors in diet as prediſpoſing to nervous diſeaſes, our author obſerves, that ſome kinds of food eaſily affect irritable and ſometimes even the ſtrongeſt nerves. He has ſeen ſeveral hyſterical women inſtantly affected by the ſmell of parſley, and in the *Journ. de Med.* a caſe is related in which this plant occaſioned convulſions. Similar effects have been produced by ſtrawberries, craw-fiſh, and even crabs eyes. When dyſpepſia has taken place to a certain degree, aliment of any kind, taken in too great a quantity, will be liable to incommode the patient. In general, ſuch as is flatulent or acid is the moſt dangerous. Of the farinaceous vegetables, potatoes, though not the moſt ſavoury, are ſpoken of as the mildeſt, and the eaſieſt of digeſtion. Maize or Turkey wheat likewiſe affords a mild farinaceous aliment. In ſome caſes, milk is the only food that agrees with the patient.

Our author points out the bad effects of wine and ſpirituous liquors, as exciting and increaſing nervous diſeaſes. He ſpeaks of Malaga, Frontiniac, and Cyprus, as the moſt innocent wines of any. He

 very very judiciously condemns the abuse of tea, and tepid liquors. Coffee has lately been spoken of as an antispasmodic, and recommended in asthma, but our author speaks of a case of spasmodic asthma, in which it constantly produced bad effects, and in several instances of morbid irritability he has generally found that it aggravated the patients sufferings. After offering his remarks on diet, M. Tissot proceeds to treat of sleep and watching, exercise and rest.

He considers sleep as a temporary palsy, during which the voluntary action of all the muscles ceases &c. As a proof of the great diminution of this action during sleep, he observes that a man who falls asleep in the open air when the thermometer is 8 or 9 degrees below 0, commonly dies; whereas a person in motion is capable of supporting a cold thirty degrees and upwards greater than this. He quotes a case from Boerhaave, of a Physician who by indulging too much in sleep, at length became quite an idiot. To the weakness induced by sleep, our author ascribes the spasmodic complaints that so often occur to nervous patients when asleep. He knows a person of a very irritable fibre, who frequently, and more particularly at the approach of rainy weather, experiences violent convulsive sensations

ſenſations in his ſtomach and breaſt, and ſometimes in every part of his body, juſt as he is going to ſleep; and M. Martin of Lauſanne in the *Mem. de l'Acad. des Sciences* for 1732, ſpeaks of a patient who was troubled with convulſions whenever he continued in bed after his firſt ſleep.

Exercife may in general be conſidered as tending to prevent and to cure rather than to occaſion nervous diſeaſes; ſometimes however, when carried to exceſs, it lays the foundation of debility and ſpaſm. Inſtances of ſuch an effect may be met with in practical writers, and our author once had occaſion to ſee a robuſt young man who was attacked, from this cauſe, with ſevere pain in every part of his body, and violent cramps in his hands and legs: too much watching ſeems to produce nearly the ſame effects as too much bodily exerciſe, by exhauſting the powers of the brain, and thus preventing that ſupply of animal ſpirits which is ſo eſſential to life and health. Our author himſelf, at the age of nineteen, by ſix weeks almoſt continually watching, deprived himſelf of that quiet, healthy ſleep which he before conſtantly experienced, but which has never perfectly returned ſince.

M. Tiſſot treats very fully of excretions and retentions. Of all evacuations that of the ſeminal fluid is the moſt baneful; but he paſſes ſlightly over the effects of immoderate venery, having deſcribed them fully in his work *de Onania.* He obſerves, that evils may likewiſe ariſe from too long abſtinence from venery, but that they occur more frequently to women than to men. He quotes a paſſage from Stegman's *Hiſtor. Epid. Mansfeld.* ann. 1698, to prove that the furor uterinus is ſometimes epidemic: Stegman's words are, "in June, July, and Auguſt, mania, melan- "cholia, & furor uterinus were epidemic in this "place; I myſelf ſaw eighteen inſtances of the "latter." The good effects of marriage, added M. Tiſſot, in ſome women ſubject to nervous complaints, prove the exiſtence of this cauſe. Schmid (*Medicina Septentrion.* tom. 2.) had the care of an hyſterical woman, who after having been bled 176 times, and taken a variety of remedies without ſucceſs, was at length cured by marriage; the ſame author, however, afterwards adds another caſe of hyſteria in which this remedy was of no effect; and M. Tiſſot is convinced that its good effects have been too much exaggerated, and that it has often been productive of miſchief.

[ *To be continued.* ]

